# Risk of Developing Melanoma With Systemic Agents Used to Treat Psoriasis: A Review of the Literature

**DOI:** 10.1177/12034754211038509

**Published:** 2021-08-15

**Authors:** Amy Semaka, Thomas G. Salopek

**Affiliations:** 1 3158 Faculty of Medicine & Dentistry, University of Alberta, Edmonton, AB, Canada; 2 Division of Dermatology, Department of Medicine, University of Alberta, Edmonton, AB, Canada

**Keywords:** psoriasis, melanoma, skin cancer, biologics, immunosuppressives

## Abstract

**Background:**

Psoriasis is a chronic inflammatory skin disease induced by autoimmune-like dysregulation of the immune system. Treatment options have drastically evolved in recent years, and treatment advances that target specific cytokines and other molecules involved in dysregulation have had a profound effect in controlling the disease.

**Objective:**

We reviewed the literature to assess the risk of developing melanoma with conventional therapies and newer agents used to treat psoriasis.

**Methods:**

A comprehensive literature search using Medline (via Ovid) and Embase was conducted.

**Results:**

The majority of studies reviewed reported insignificant results. Potential risk for melanoma was identified for only 3 out of 15 anti-psoriatic treatments analyzed: adalimumab (relative risk 1.8, 95% CI 1.06-3.00), etanercept (relative risk 2.35, 95% CI 1.46-3.77) and infliximab (Empirical Bayes Geometric Mean 7.90, 95% CI 7.13-8.60). The confidence intervals provided are from prior studies. There are not enough collective data on newer agents to make any conclusions on risk.

**Conclusions:**

We were unable to identify any substantial risk for developing melanoma due to the use of anti-psoriatic treatments. Until additional long-term registry data become available, it would be prudent to continue screening patients with psoriasis at baseline and periodically for melanoma when these agents are used.

## Introduction

In recent years, there has been a revolution in the treatment of psoriasis such that we now have a plethora of therapies at our disposal. An improved understanding of the immune system and its role in the pathogenesis of psoriasis has led to the development of more targeted therapies with significant improvements in disease control. For most patients with psoriasis, we are now able to achieve remission or near remission with biologics or small molecule agents.

Worldwide the incidence of melanoma has been increasing at a faster rate than almost all other cancers.^1^ The lifetime risk for developing this cancer varies from about 1:39 in the United States to 1:56 in Canada.^1,2^ Numerous risk factors have been implicated, including excessive ultraviolet (UV) exposure from the sun or tanning beds, fair skin that easily burns, a positive family history, having more than 50 moles, and a history of severe sunburns. A direct relationship between UVB exposure and melanoma has been demonstrated, with a 10% increase in average annual UVB irradiation correlating with a 19% increased risk of melanoma.^1^ DNA mutations are at the core of this relationship, and UVA irradiation may also cause similar effects. Prevention and early detection of melanoma are the most significant factors in survival, but detection still gets overlooked as evidenced by low levels of screening in some populations.^3^

The prevalence of psoriasis is estimated to be approximately 2% of the total population worldwide.^4^ In light of the lifetime risk for melanoma, a significant number of patients with psoriasis will develop this cancer. Excluding phototherapy, which has been shown to increase the risk of melanoma, there is no definite association between psoriasis and melanoma.^5-8^ What is unknown is whether the agents used to treat psoriasis increase the risk for developing this cancer. To address this issue, we reviewed the literature for conventional treatments, small molecule drugs and biologics used in the management of psoriasis to assess whether or not they increase the possibility of developing melanoma.

## Methods

**Figure 1:**
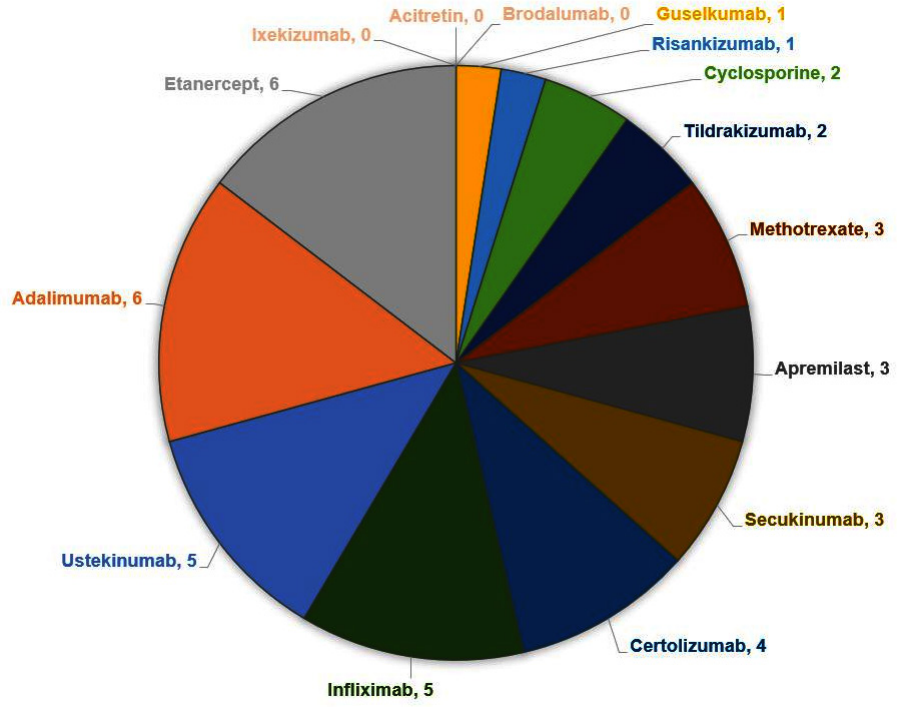
Breakdown of articles included.

This was a comprehensive literature review. We collaborated with a professional librarian for assistance with our search. The literature search was conducted in Medline (via Ovid) and Embase and included 15 treatments that are approved for psoriasis in North America. The keywords used were the 15 generic drug names and their corresponding brand names (acitretin or Soriatane^®^, apremilast or Otezla^®^, cyclosporine, methotrexate, adalimumab or Humira^®^, certolizumab or Cimzia^®^, etanercept or Enbrel^®^, infliximab or Remicade^®^, ustekinumab or Stelara^®^, guselkumab or Tremfya^®^, risankizumab or Skyrizi^®^, brodalumab or Siliq^®^, ixekizumab or Taltz^®^, secukinumab or Cosentyx^®^, tildrakizumab or Ilumya^®^), combined with psoriasis and melanoma. A breakdown of the articles chosen is provided (Figure 1).

One author searched for articles and subsequently reviewed them to ascertain their relevance. Titles and abstracts were screened and the following were excluded: non-English, irrelevant, duplicates, conference proceedings, treatments no longer on the market, or if the publication was inaccessible (ie, could not be obtained through interlibrary loans or was not available online). All types of studies were examined and any year of publication. If articles discussed malignancies in general but did not specifically report on melanoma, they were excluded. Reference lists were reviewed as needed to retrieve additional articles and ensure available data were adequately collected.

## Results

A total of 550 possible articles were identified in our search. Of those, 35 individual articles specific to the 15 treatments for psoriasis were selected for inclusion (Supplemental Table S1).

### Conventional Treatments

Of the conventional treatments investigated, cyclosporine was not found to have an increased risk of melanoma.^9,10^ Methotrexate was found to have a possible increased risk in one trial (Kaplan Meier estimate for 5-year risk 0.48%, 95% CI 0.43% to 0.53%),^11^ but this was refuted in later studies.^12,13^

### Small Molecule Drugs

Apremilast was the only small molecule drug investigated. A case report was found that described the recurrence of a patient’s melanoma coinciding with the clearance of their psoriasis, shortly after they were started on apremilast.^14^ Other studies did not report any melanoma cases.^15,16^

### TNF-Α Inhibitors

Adalimumab had a significant relative risk (RR) for melanoma in one retrospective study (1.8, 95% CI 1.06-3.00), as did etanercept (2.35, 95% CI 1.46-3.77).^17^ Infliximab had a significant safety signal, reported as an empirical Bayes geometric mean (EBGM) of 7.90 (95% CI 7.13-8.60) in the same study. It is worth noting that this study included multiple chronic inflammatory conditions and was not limited to patients with psoriasis. There was one case report of a patient who developed a melanoma recurrence 4 weeks into psoriasis treatment with etanercept,^18^ and another case of a patient with no apparent risk factors who developed melanoma after being on etanercept for 5 years.^19^ Three case reports for infliximab were found: one patient developed nodular amelanotic melanoma 24 months into treatment with infliximab and methotrexate^20^; one patient developed nodal melanoma metastasis under infliximab therapy for 12 months^21^; another patient developed cerebral melanoma metastasis after being treated with infliximab for 13 years.^22^ Other studies of various duration demonstrated no increased risk or did not report melanoma cases with adalimumab,^23-27^ etanercept^28-30^ and infliximab.^31^ There was neither a safety signal, nor any reports of melanoma, in studies of certolizumab.^17,32-34^

### IL-12/23 Inhibitors

Safety analyses did not report significant risk of melanoma for ustekinumab.^35-37^ Two psoriasis patients with histories of melanoma had no recurrence while on ustekinumab.^22,38^

### IL-23 Inhibitors

Short-term trials did not report significant risk of melanoma for guselkumab,^39^ risankizumab,^40^ or tildrakizumab.^41,42^

### IL-17 Inhibitors

There were no data found for either brodalumab or ixekizumab. There were no significant findings for secukinumab.^43,44^ One psoriasis patient with a history of melanoma had no recurrence while on secukinumab.^22^

## Discussion

This review of existing literature on systemic agents used to treat psoriasis brings into light the uncertainty of melanoma risk. Even despite historical psoriasis treatment with UV light, there does not appear to be a significant melanoma risk with our currently available treatment options. Logically, newer agents (IL-12/23 inhibitors, IL-23 and IL-17 inhibitors) have yet to amass enough use in broad populations for any potential risk of melanoma to be identified. Numerous reviews comparable to this 1 have been published in the past, with similar findings and conclusions.^45^ In 2014, a retrospective U.S. database analysis demonstrated that the proportion of melanoma cases in patients on non-biologic treatment, adalimumab, infliximab, and etanercept were not significant, nor were the rates significantly different from those found in the overall psoriasis population.^46^ In 2015, a nationwide registry analysis in Germany found no relevant differences between any treatments used for psoriasis with respect to melanoma rates, and the rates were very low.^47^ Another nationwide database analysis in France found no signal for melanoma with TNF-α inhibitor use across various inflammatory conditions, although they postulated this could have been due to a lack of power of their sample.^48^

In 2017, a cohort analysis from Northern California found that melanoma rates in psoriasis patients were not significantly different between biologically-exposed and unexposed groups,^49^ and another study from Southern California indicated there was no increased risk for melanoma in psoriasis patients on systemic agents or biologics as compared to patients on topical treatments.^8^ A large case-control registry analysis across North and South America and Europe noted that melanoma was one of the 5 most frequently occurring malignancies in patients on systemic psoriasis treatments but the incidence rate was 0.06 per 100 patient-years (95% CI 0.04-0.09).^50^ A literature review from 2018 concluded that the data regarding the risk of melanoma in psoriasis patients treated with TNF-α inhibitors is conflicting.^51^

More recently, a systematic review and meta-analysis of patients with inflammatory diseases being treated with biologics (mostly TNF-α inhibitors) stated that clinically important increases in melanoma risk cannot be ruled out.^52^ The hazard ratio (HR) for psoriasis was 1.57 (95% CI 0.61-4.09). Only 1 study included in this analysis pertained to psoriasis. Another systematic review and meta-analysis revealed no increased risk of cancer [overall] in patients with psoriasis treated with biologic agents.^53^ Interestingly, data from other chronic inflammatory conditions such as rheumatoid arthritis and inflammatory bowel disease studies are not entirely conclusive regarding the risk of melanoma with systemic or biologic treatment, including TNF-α inhibitors.^54-58^

It is worthwhile noting that TNF-α’s pleiotropic roles in the biology and evolution of cancer have only recently begun to be appreciated, in the last decade or so.^59^ Numerous articles over the years have incriminated TNF-α inhibitors as contributing to malignancy, but many articles suggest it has a pro-tumor effect in melanoma.^60-63^ It has been remarked that perhaps instead of trying to increase TNF tumor levels, blocking TNF-α may unveil a viable strategy to boost immune checkpoint inhibitor (ICI) response in patients with metastatic melanoma.^63^ In a mouse melanoma model, using an ICI alone allowed for the regression of 20% of tumors, but combining it with a TNF-α inhibitor induced the regression of 75% of tumors.^63^

The major limitation with this review is our lack of scrutiny regarding the types of patients included in our selected articles. Because this was a literature review, critical appraisal of included studies was out of scope. We recognize that not all studies address confounding factors that would increase the risk of melanoma, such as tanning bed use or a personal or family history. A trial population with a high prevalence of melanoma could conceal any possible association of drug and melanoma risk and trial duration can influence the prospect of risk, with longer trials potentially enabling the emergence of melanoma over a greater period of time. As previously mentioned, some treatments are still in their infancy on the market (ie, brodalumab, guselkumab, ixekizumab, risankizumab, tildrakizumab), and there has not been enough time to adequately capture a risk. Furthermore, combining data from multiple chronic inflammatory conditions can blur the interpretation of risk, specifically when a breakdown for each inflammatory condition investigated is not provided as is the case with one article we included.^17^

The primary strength of our literature review is the comprehensive examination of 15 individual psoriasis treatments spanning both older and new and from a multitude of study types that were reported across the world. We provide a pertinent summary of existing literature that encompasses a breadth of results. Our findings may allow clinicians to provide their psoriasis patients with favorable drug treatments that improve their disease control and better yet, their quality of life, while not diminishing the obvious need to continue monitoring patients for melanoma. Fortunately, psoriasis patients likely have heightened screening with routine dermatology visits. As their psoriasis clears, it may uncover melanoma and clinicians should bear in mind this optimal time to perform a thorough skin exam. Patient education and teaching self-exams should not be underestimated as well. Increased awareness is indispensable for both clinicians and patients, especially as drug development continues to evolve and longer-term epidemiological data accumulate.

## Conclusion

The current evidence for melanoma risk in psoriasis patients being treated with systemic agents is unsubstantiated. The development and progression of melanoma is highly complex and likely depends on the interaction between a number of different factors. Additional studies investigating the recurrence of melanoma in patients with a current or past diagnosis and using systemic or biologic agents would be valuable to better understand any possible relationship between the two. Additionally, studies that expose the mechanism of action of various treatments and their implications for melanoma development and progression would add to our understanding of these relationships. The overall findings from this literature review are inconsistent but a risk apart from drug therapy can never entirely be excluded, and with that, one thing is for certain: we should continue screening our patients for the presence of melanoma, to foster early detection when the chances of survival are at their highest.

## Supplemental Material

Online supplementary file 1 - Supplemental material for Risk of Developing Melanoma With Systemic Agents Used to Treat Psoriasis: A Review of the Literature Click here for additional data file. Supplemental material, Online supplementary file 1, for Risk of Developing Melanoma With Systemic Agents Used to Treat Psoriasis: A Review of the Literature by Amy Semaka and Thomas G. Salopek in Journal of Cutaneous Medicine and Surgery
