# Supplementary material for: Risk of Developing Melanoma With Systemic Agents Used to Treat Psoriasis: A Review of the Literature
Source: J Cutan Med Surg. 2021 Aug 15;26(1):87–92. doi: 10.1177/12034754211038509 (PMC8750137; doi:10.1177/12034754211038509)
Supplement: Online supplementary file 1 - Supplemental material for Risk of Developing Melanoma With Systemic Agents Used to Treat Psoriasis: A Review of the Literature [file sj-docx-1-cms-10.1177_12034754211038509.docx]

**Supplemental Material**

**Risk of developing melanoma with systemic agents used to treat psoriasis: a review of the literature**

Amy Semaka, BScPharm, PharmD^1^, Thomas G. Salopek, MD, FRCPC^2^

^1^Faculty of Medicine & Dentistry, University of Alberta, Edmonton, Alberta, Canada

^2^Division of Dermatology, Department of Medicine, University of Alberta, Edmonton, Alberta, Canada

| **Table 1. Psoriasis Studies** | | | | | | |
| --- | --- | --- | --- | --- | --- | --- |
| **Drug** | **Study Design** | **Duration** | **Patients/**  **Patient-Years** | **Findings** | **Risk Assessment** | **Reference** |
| ***Conventional Therapies*** | | | | | | |
| Acitretin (Soriatane^®^) | No data found | | | | | |
| Cyclosporine | Multicenter cohort study | 5 years | 1,252 patients | 2 reports of melanoma | NR | 9 |
|  | Literature review | 25 years of data | >1,700 patients | “The notion that [cyclosporine] may contribute to the development and spread of melanoma is not supported” | NR | 10 |
| Methotrexate (MTX) | Retrospective cohort study (Sweden) | 9 years of data | 101,966 patients | Small but significant increase in melanoma with MTX-exposed patients | KM estimate for 5-year risk: 0.48% (95% CI 0.43-0.53%) in MTX-exposed, vs 0.41% (95% CI 0.39-0.43%) in MTX-unexposed | 11 |
|  | Retrospective cohort study (Sweden) | 9 years of data | 101,966 patients | Could not prove a reproducible dose-response relationship between melanoma risk and MTX dose | NR | 12 |
|  | Nested case-control study (Sweden) | 6 years of data | 395 patients | No association between MTX exposure (ever use) and melanoma was observed | OR 1.0 (95% 0.8-1.3) | 13 |
| ***Small Molecule Drugs*** | | | | | | |
| Apremilast (Otezla^®^) | Multicenter phase III RCT | 52 weeks | 413 patients | “Exposure-adjusted IR for malignancies in pooled analyses was comparable between apremilast and placebo” | NR | 15 |
|  | Multicenter phase IIIb RCT | 52 weeks | 250 patients | No reports of melanoma | NR | 16 |
|  | Case report | 4 months | 1 patient | 32-year-old male with a history of 2 previous melanomas, but disease-free for >3 years; within ~4 months of apremilast treatment for psoriasis, he developed recurrence of his melanoma in keeping with cutaneous metastasis (stage IVa) | NR | 14 |
| ***TNF-α Inhibitors*** | | | | | | |
| Adalimumab (Humira^®^) | Comprehensive safety analysis | Mean duration 19.3 patient months | 1,188 patients, 370.5 patient-years | 9 reports of melanoma | 0.2/100 PY | 24 |
|  | Comprehensive safety analysis | 12 years of data | 3,010 patients, 5,062 patient years | 8 reports of melanoma from psoriasis trials | SIR 4.37 (95% CI 1.89-8.61) | 23 |
|  | Retrospective study (USA) | Data from date of FDA approval through Aug 2012 | 237 patients | Significant risk and safety signal found; results were across multiple chronic inflammatory diseases | EMR database: RR 1.8 (95% CI 1.06-3.00);  FDA Adverse Events Reporting System database: EBGM 2.49 (95% CI 2.19-2.83) | 17 |
|  | Worldwide observational study | 5 years | 6,059 patients, 19,243 patient-years | 10/178 reports of malignancy were melanoma | <0.1/100 PY | 25 |
|  | Comprehensive safety analysis | Mean duration 17.5 months | 3,727 patients, 5,430 patient-years | Updated analysis; 10 reports of melanoma | 0.2/100 PY | 26 |
|  | Systematic review | Exposure ranged from 88-19,243 PY | Range of 129-6,059 patients | Rates of melanoma were low | ESPRIT registry: <0.1/100 PY  PsoBest registry: <0.2/100 PY | 27 |
| Certolizumab (Cimzia^®^) | Retrospective study (USA) | Data from date of FDA approval through Aug 2012 | 13 patients | Across multiple chronic inflammatory diseases and using the FDA Adverse Events Reporting System database, no safety signal for melanoma was detected | NR | 17 |
|  | Literature review | 96 weeks | 393 patients | No reports of melanoma | NR | 32 |
|  | Multicenter phase III RCT | 48 weeks | 559 patients | No reports of melanoma | NR | 33 |
|  | Multicenter phase III RCT | 48 weeks | 461 patients | No reports of melanoma | NR | 34 |
| Etanercept (Enbrel^®^) | Case report | 4 weeks | 1 patient | 61-year-old woman with history of stage IB melanoma 6 years prior developed recurrence 4 weeks after starting etanercept | NR | 18 |
|  | Post-hoc cohort analysis | 4 years | 506 patients | No reports of melanoma | NR | 28 |
|  | Comprehensive safety analysis | 12 weeks (short-term) & 144 weeks (long-term) | 1,965 (short-term), 4,410 (long-term) | No reports of melanoma in either analysis | NR | 29 |
|  | Literature review | 3,966 PY of exposure | 13,877 patients | Across all indications, post-marketing surveillance showed lower incidence rate than that in the general population | SIR 1.74 (95% CI 0.83-3.20);  IR 12.6 vs 19.6/100,000 PY | 30 |
|  | Retrospective study (USA) | Data from date of FDA approval through Aug 2012 | 347 patients | Significant risk and safety signal found; results were across multiple chronic inflammatory diseases | EMR database: RR 2.35 (95% CI 1.46-3.77);  FDA Adverse Events Reporting System database: EBGM 2.49 (95% CI 2.24-2.76) | 17 |
|  | Case report | 5 years | 1 patient | 79-year-old male on etanercept for 5 years developed a new superficial spreading melanoma with no apparent risk factors | NR | 19 |
| Infliximab (Remicade^®^) | Case report | 24 months | 1 patient | 43-year-old male developed a nodular amelanotic melanoma 24 months into treatment with infliximab and methotrexate | NR | 20 |
|  | Case report | 12 months | 1 patient | 53-year-old woman developed nodal melanoma metastasis under infliximab therapy for 12 months | NR | 21 |
|  | Retrospective study (USA) | Data from date of FDA approval through Aug 2012 | 434 patients | Significant safety signal found; results were across multiple chronic inflammatory diseases | FDA Adverse Events Reporting System database: EBGM 7.90 (95% CI 7.13-8.60) | 17 |
|  | Case report | 13 years | 1 patient | 53-year-old woman being treated with infliximab for 13 years was diagnosed with a cerebral melanoma metastasis | NR | 22 |
|  | Multicenter retrospective study (France) | Mean duration 8.5 years | 43 patients | No reports of melanoma | NR | 31 |
| ***IL-12/23 Inhibitors*** | | | | | | |
| Ustekinumab (Stelara^®^) | Pooled safety analysis of phase II/III trials | 3 years | 3,219 patients | 3/27 skin malignancies were melanoma | NR | 35 |
|  | Pooled safety analysis of phase II/III trials | 3 years | 3,117 patients | 2 reports of melanoma | NR | 36 |
|  | Comprehensive safety analysis | 5 years | 3,117 patients, 8,998 patient-years | 6 reports of melanoma (1 invasive, but in a patient with pre-existing melanoma) | SIR 1.42 (95% CI 0.52-3.09) | 37 |
|  | Case report | 7 years | 1 patient | Patient with history of superficial melanoma treated for 7 consecutive years on ustekinumab with no relapse | NR | 38 |
|  | Case report | 13 months | 1 patient | 53‐year‐old woman with recent history of cerebral melanoma metastasis treated with sequential secukinumab (10 months) and ustekinumab (13 months) with no relapse | NR | 22 |
| ***IL-23 Inhibitors*** | | | | | | |
| Guselkumab (Tremfya^®^) | Pooled safety analysis from two phase III RCTs | 100 weeks | 1,829 patients | 1 patient developed melanoma after crossover to guselkumab from adalimumab | NR | 39 |
| Risankizumab (Skyrizi^®^) | Pooled safety analysis | 16 weeks & up to 40 months | 2,232 patients | 1 patient developed melanoma in situ; melanoma was one of the “most frequent” skin tumors reported in the longer term but only 2 cases | <0.1/100 PY | 40 |
| Tildrakizumab (Ilumya^®^) | Pooled data from two worldwide phase III RCTs | 28 weeks | 772 and 1,090 patients | No reports of melanoma | NR | 41 |
|  | Literature review | 52 weeks | Range of 77-1,090 patients | 1 report of melanoma | NR | 42 |
| ***IL-17 Inhibitors*** | | | | | | |
| Brodalumab (Siliq^®^) | No data found | | | | | |
| Ixekizumab (Taltz^®^) | No data found | | | | | |
| Secukinumab (Cosentyx^®^) | Pooled safety analysis of phase II/III trials | Ranged from 32-338 days | 3430 patients, 2,725 patient years | 4 reports of melanoma (all of which were confounded with other risk factors) | NR | 43 |
|  | Literature review | 36-52 weeks | Range of 43-1,306 patients | Excluding NMSC, melanoma was one of the most frequently reported malignancies (n=4) | NR | 44 |
|  | Case report | 10 months | 1 patient | 53‐year‐old woman with recent history of cerebral melanoma metastasis treated with sequential secukinumab (10 months) and ustekinumab (13 months) with no relapse | NR | 22 |
| *EBGM, empirical Bayes geometric means; CI, confidence interval; IL, interleukin; IR, incidence rate; KM, Kaplan-Meier; NMSC, non-melanoma skin cancer; NR, not reported; OR, odds ratio; PY, patient years; RCT, randomized controlled trial; RR, relative risk; SIR, standardized incidence rate; TNF, tumor necrosis factor* | | | | | | |
